# Influence of Cooking Technique on Bioaccessibility of Bioactive Compounds in Vegetable Lentil Soup

**DOI:** 10.3390/foods13152405

**Published:** 2024-07-29

**Authors:** Sofía Vargha, Marta Igual, Marcelo Miraballes, Adriana Gámbaro, Purificación García-Segovia, Javier Martínez-Monzó

**Affiliations:** 1 Food Department, School of Nutrition, Universidad de la República (UdelaR), Montevideo 11800, Uruguay; svargha@nutricion.edu.uy; 2 Food Technology Department, Universitat Politècnica de València, 46022 Valencia, Spain; marigra@upvnet.upv.es (M.I.); pugarse@tal.upv.es (P.G.-S.); xmartine@tal.upv.es (J.M.-M.); 3 Food Science and Technology Department, School of Chemistry, Universidad de la República (UdelaR), Montevideo 11800, Uruguay; mmiraballes@fq.edu.uy

**Keywords:** in vitro digestion, bioaccessibility, bioactives, cooking methods, soup

## Abstract

Vegetables and legume soups contain various essential and bioactive constituents such as vitamin C, carotenoids, and phenolics. Antioxidant activity characteristics related to those compounds are well known to contribute profusely to human health. The cooking technique affects the bioavailability of nutrients and bioactive compounds, making it crucial to explore optimal alternatives to maximize them. The objective of this study was to explore the influence of different cooking techniques (boiling, pressure cooking, sous-vide, and cook-vide) on the physicochemical properties and bioactive characteristics of a ready-to-eat vegetable lentil soup. For this, the bioaccessibility of those compounds was assessed through an in vitro simulated gastrointestinal methodology. The firmness of vegetables was established to define treatments’ cooking times, allowing subsequent comparison of the nutritional and functional properties of the soups. The color of vegetables was also evaluated as a quality parameter, which contributed to providing a global vision of the process impact. The results revealed that in vitro digestion (IVD) caused a decrease in all bioactive compound determinations for all cooking treatments of up to 72% for total phenols, 92% for lycopene, 98% for carotenoids, and 100% for vitamin C. Additionally, the antioxidant activity of the soups after thermal treatment improved up to 46% measured by the DPPH method. This study emphasizes the importance of considering the digestion process in the selection of the most adequate cooking technique. After IVD, traditional cooking (boiling) reached the maximum total carotenoid and lycopene contents; cook-vide and pressure-cooking techniques provided the highest total phenol content, showing these three techniques to have the maximum antioxidant capacity.

## 1. Introduction

The current consumer is informed regarding health and increasingly demands products or preparations of higher nutritional quality that retain their sensory characteristics and, ideally, require a minimum processing or reconstitution time [1,2,3]. Convenience foods such as ready-to-cook and ready-to-eat meals act as the optimal solution for these consumers; however, the major challenge is providing a completely balanced diet [4].

An association between a healthy diet and reduced risks of noncommunicable diseases is established with adequate vegetable consumption [5]. Vegetables contribute to a healthy and well-balanced diet by providing vitamins and phytochemicals associated with a reduced risk of cancer, diabetes, and cardiovascular diseases [6]. Nutritional variety plays a crucial role in maintaining optimal health [7]. Consuming a diverse range of foods provides essential vitamins, minerals, and antioxidants necessary for proper bodily function. Conversely, a lack of dietary diversity can lead to nutrient deficiencies and increase the risk of chronic diseases [8]. By promoting nutritional diversity and emphasizing proper dietary follow-up, we can enhance overall well-being [9].

Vegetable soup is a staple diet for billions of people worldwide. Vegetable soup exists in many traditional varieties and is generally consumed for health and nutritional benefits [10,11]. This food product is nutritionally a great option, as it contains a high nutrient density vital for physical and mental health [12,13]. However, adding protein, mineral, and vitamin sources could improve its nutritional quality by mixing vegetables and legumes [13].

Vegetable and legume soups contain various essential and bioactive constituents, such as vitamin C (vitC), carotenoids, and phenolics, and their antioxidant activity characteristics are well known to contribute profusely to human health [14,15].

Nutrition quality does not only include compositional parameters but should also include the gastrointestinal breakdown leading to optimal biological functionality [16,17]. The evaluation of bioactive compounds that are released from the food matrix before being absorbed by the small intestine is called bioaccessibility [18,19,20]. In vitro simulated gastrointestinal methodology (in vitro digestion, IVD), being an effective method to measure bioaccessibility, simulates the digestion process, enabling nutrient measurements on final extracts [21]. The advantages of this method include it being rapid, safe, and not having the same ethical restrictions as in vivo methods, which is why it is currently being extensively used [22]. In particular, an in vitro, general, standardized, and practical static digestion method based on relevant conditions is the one proposed by the COST INFOGEST network [23]. This protocol has been extensively used for various purposes, including evaluating the bioaccessibility of some bioactive compounds from the vegetable matrix [24]. However, there is still a need to investigate the digestibility and bioaccessibility of food constituents to understand their fate in the human body [25].

Cooking has the aim of prolonging storage time, providing an appetizing aspect, aroma, and flavor, reducing micro-organism charges, deactivating antinutrients or toxic substances naturally present in the raw product, and improving digestibility [26,27]. In vegetables, cooking causes starch gelatinization and β-elimination of pectic substances, thereby increasing fiber digestibility, changing organelles in the cytoplasm, and causing protoplasmic structure and organization breakdown. Moreover, the destruction of the cell wall and membrane during cooking leads to a change in the bioaccessibility and bioavailability of vitamins, minerals, phytochemicals, and so on [4]. However, the cooking process also causes the degradation of thermolabile vitamins and phytochemicals, the destruction of some essential amino acids, and the leakage of mineral salts and vitamins in cooking water [28].

Generally, the most common method to cook vegetables and legumes is boiling (at atmospheric pressure), also known as traditional cooking (TC) [27]. TC is known to be a harsh treatment that contributes to the loss of flavor and color, and many heat-labile compounds are lost or partially destroyed when leached into the cooking water.

Another popular cooking method is pressure cooking (PC), which uses water or other liquid in a sealed pot, usually a pressure cooker [29]. Although the pressure inside the pot is higher than atmospheric pressure, the cooking temperature is over 100 °C, and the cooking time is reduced compared with the TC method.

Vacuum cooking has gained attention as an alternative cooking method in terms of application at low temperatures, short processing times, a nonoxygen environment, and better protection of the nutritional value and texture of the food [30]. Cook-vide (CV) (vacuum steam cooking) consists of cooking the product under conditions of subatmospheric pressure (vacuum) [31]. Conversely, the sous-vide (SV) method requires raw food to be preliminarily vacuum-sealed in heat-stable plastic pouches; afterward, the food is cooked by introducing it to hot water at low temperatures using precise, controlled heating [32]. Sous-vide cooking offers several advantages over traditional methods in terms of cost-effectiveness [3]. It is energy-efficient due to precise temperature control, resulting in minimal energy consumption [28,29]. Additionally, sous-vide cooking minimizes food waste by maintaining uniform doneness and reducing cooking losses [28,29]. Studies conducted by Iborra et al. [33] evaluated the structural, nutritional, and sensory features of vegetables cooked with sous-vide treatment and traditional cooking. This study shows that traditionally cooked samples suffered more loss of turgor than the other treatments. In addition, an increase in gaps between cell walls is observed in traditional cooking. According to results reported by Iborra et al. [33], sous-vide-treated tissues were subjected to a pressure that favored the better conservation of their structure (cell wall contact) and the presence of some organelles [33].

The nutritional and functional value of food varies depending on the ingredients used and nutrient losses recorded during the cooking process [34]; the preservation and bioaccessibility of nutrients are highly dependent on the latter [15,21,29,35]. Consequently, the effect of cooking on the nutritional, functional, and sensory qualities of vegetables and legumes is a topic of increasing interest [6]. Many studies have evaluated the impact of cooking methods (i.e., pressure, boiling, steaming, vacuum, and microwave cooking) on the nutrients of vegetables and legumes [5,29,35]. However, to the authors’ knowledge, there are still no studies evaluating the impact of different cooking techniques on the bioaccessibility of vegetables and legumes, so it is relevant to investigate this aspect.

Given this background, the present study aims to evaluate and compare the influence of cooking techniques (boiling, PC, SV, and CV) on the texture, color, and bioactive compounds’ bioaccessibility in ready-to-eat vegetable lentil soup.

## 2. Materials and Methods

### 2.1. Raw Materials

Fresh vegetables and legumes were used, with the addition of distilled water. Pumpkin, carrot, pepper, onion, celery, and lentils were purchased from a local company (Mercadona, Valencia, Spain) 1 day before the experiments. Vegetables were in fresh, raw form, except for pumpkin, which was peeled and packaged in a plastic container. Vegetables were all refrigerated at 5 °C in the store and kept stored at the same temperature; lentils were packaged in a plastic bag and stored at 25 °C.

### 2.2. Soup Formulation

The vegetables and lentil soup ingredients and proportions are water (66%), pumpkin (9%), carrot (8%), lentil (8%), pepper (3%), onion (3%), and celery (3%). The formulation was established according to Uruguayan preferences in a previous qualitative study with consumers.

The processing of the fresh raw vegetables involved peeling the onion and carrots; the pumpkin was already prepeeled; and the pepper and celery were cleaned under running water. The material prepared was cut into cubic pieces using a manual metal cutter: pepper, onion, and celery were cut into small pieces (5 mm × 5 mm × 5 mm), carrots into medium pieces (5 mm × 5 mm × 10 mm), and pumpkins into large pieces (10 mm × 10 mm × 10 mm).

### 2.3. Soup Preparation

The cut vegetables and lentils were weighed and transferred to the cooking container (pot or bag according to the cooking technique), and then distilled water was added. Subsequently, the container was sealed when appropriate, and the sample was heated. After each treatment, the ready-to-eat soup product is finished. Subsequently, the product is rapidly cooled, frozen, and lyophilized for future analyses.

Four cooking techniques were applied in the study: boiling (traditional cooking, TC), PC, and two vacuum-cooking treatments: SV and CV. Table 1 shows some characteristics of the cooking method used in this study.

Cooking conditions were performed based on the methods by Rondanelli et al. [27], Iborra-Bernad et al. [33], Iborra-Bernad et al. [36], and Rinaldi et al. [37], with minor adjustments. The cooking conditions were established with a set temperature and varying cooking times, according to the studies mentioned before. As this study aimed to analyze soups produced with different techniques while cooked to a similar degree, the final firmness of pumpkins and carrots was used as a standard to establish comparisons. The selected final firmness values were close to 0.7 and 1.9 N (in phloem tissue) for pumpkins and carrots, respectively, based on studies by Iborra-Bernad et al. [33] and Rinaldi et al. [37]. For practical criteria, the shorter time process was considered the most adequate, ensuring all ingredients were completely cooked at the end of the treatment.

For TC, 100 °C of temperature was applied and measured using a digital thermometer (model Testo 108, Testo AG, Lenzkirch, Germany). TC was performed using a stainless-steel-covered saucepan heated by an electric cooktop for 2 min and uncovered for the rest of the set time.

The PC method was performed using a pressure cooker (Omni Perfect, BEEM, Stapelfeld, Germany), set at 85 kPa overpressure and heated by an electric induction cooktop.

For the SV treatment, the sample was vacuum-sealed (91% vacuum) in heat-resistant polyethylene pouches (Cryovac Sealed Air Co., Barcelona, Spain) using a vacuum-packaging machine (S-220 MP, VAC-STAR AG, Sugiez, Switzerland). The cooking treatment was conducted at atmospheric pressure, 90 °C, in a water bath (GD 120, Grant Instruments, Cambridge, UK).

The CV method was performed using a cooking device equipped with a lid for vacuum cooking (Gastrovac^®^, International Cooking Concepts, Barcelona, Spain). The temperature applied was 90 °C, and the corresponding pressure was 70.2 kPa.

After the cooking treatment, all samples were rapidly cooled in a water–ice bath until reaching 25 °C. Process yield was calculated based on the weight percentage between the soup and the soup ingredients before cooking. For further determination of bioactive compounds, the samples were freeze-dried. The procedure consisted of mixing the soup, placing it in trays, freezing it at −45 °C for 24 h (Vertical Freezer, CVF450/45, Ing. Climas, Barcelona, Spain), and then drying it in a Lioalfa-6 Lyophyliser (Telstar, Spain) at −56.6 °C and 2600 Pa for 48 h. Subsequently, grinding of the freeze-dried samples was applied (Minimoka, Taurus, Lleida, Spain) to obtain powder, which was homogenized for subsequent determinations.

### 2.4. Analysis

#### 2.4.1. Vegetable Mechanical and Optical Properties

In this particular soup variant, the vegetables maintain their individual form, as they are presented in small pieces. Texture analysis and color measurements were performed on individual vegetables immediately after cooking and cooling the samples.

The texture analysis was conducted only on pumpkins and carrots. The firmness of samples was measured at 25 °C by a puncture test using a Texture Analyzer TA.XT Plus (Stable Micro Systems, Surrey, UK), according to the methodology followed by Iborra-Bernad et al. [36].

The firmness test was conducted using a 2 mm diameter stainless-steel flat-head probe (TA P/2). The probe completely penetrated perpendicularly into the surface of the pieces with a penetration speed of 2 mm/s. Firmness was considered the maximum recorded force during the puncture test. One measurement for each vegetable was performed for each piece; in carrots, the measurement was performed in the phloem. A total of six measurements were analyzed for each treatment. Data were collected and analyzed using Texture Exponent Software 6.2 (Stable Micro Systems, Godalming, UK).

The color analysis between raw and cooked samples was conducted only on pumpkins and carrots. The color was measured using a TS7030 portable spectrocolorimeter (Shenzhen ThreeNH Technology, Shenzhen, China). CIE-L*a*b* coordinates were obtained using a D65 illuminant and a 10° observer as the reference system. Standard whiteboards and blackboards were used for calibration. Registered parameters were L* (brightness), a* (greenness/redness), and b* (blueness/yellowness). Total color differences (ΔE*ab, Equation (1)), hue (h* ab, Equation (2)), and chroma (C* ab, Equation (3)) were calculated using the following equations:

(1) $$\Delta E^{*}\mathrm{ab}\;=\sqrt{{\Delta L}^{*2}+{\;\Delta a}^{*2}+{\;\Delta b}^{*2}}$$

(2) $$h^{*}\mathrm{ab}\;=\;\arctan(\frac{b^{*}}{a^{*}})$$

(3) $$C^{*}\mathrm{ab}\;=\sqrt{{\;a}^{*2}+{\;b}^{*2}}$$

The surface color at the top of the pieces of pumpkins and carrots was analyzed using eight measurements.

#### 2.4.2. Total Carotenoids and Lycopene

The analysis was conducted on lyophilized samples. The extraction of total carotenoids (TotC) and lycopene (L) was realized using a hexane/acetone/ethanol solvent mixture (50:25:25, *v*/*v*/*v*) [24,38]. For quantification, the spectrophotometric reference method of AOAC (2000) measuring absorbance at a wavelength of 446 nm (TotC) and 501 nm (L) was performed using a UV-3100PC spectrophotometer (VWR, Leuven, Belgium). The results are expressed as mg of β-carotene (Fluka-BioChemika, Buchs, Switzerland) per 100 g of dried base sample, and all samples were analyzed in duplicate.

#### 2.4.3. Total Phenols

The analysis was conducted on lyophilized samples. The method described by Igual et al. [39] and Igual et al. [24] was followed for total phenols’ determination based on the Folin–Ciocalteu method. Methanol was used as a solvent for the samples’ extraction. Briefly, the sample was mixed with methanol and centrifuged at 10,000 rpm at 4 °C for 10 min using an Eppendorf Centrifuge 5804 R (Eppendorf, Hamburg, Germany). Subsequently, 250 µL of supernatant was mixed in a 25 mL volumetric flask with 1250 µL of Folin–Ciocalteu reagent (Panreac-AppliChem, Darmstadt, Germany) and kept for 8 min in a dark place at 25 °C. Afterward, 3.75 mL of sodium carbonate aqueous solution (7.5%) was added, adjusted to the final volume, and then stored for 120 min at 25 °C. The total phenolic content (TP) was determined by absorbance at 765 nm measured using a UV–Vis spectrophotometer (UV-3100PC, VWR, Radnor, Philadelphia, PA, USA) and expressed as mg of gallic acid equivalents (Sigma-Aldrich, Darmstadt, Germany) per gram of sample. Samples were analyzed in triplicate before and after IVD.

#### 2.4.4. Antioxidant Capacity

The antioxidant capacity (AC) was assessed using the free radical-scavenging activity of the samples according to Igual et al. [39] and Igual et al. [24] based on the DPPH method (evaluation with the stable radical 2,2-diphenyl-1-picrylhydrazyl). The DPPH method has been chosen because, among the spectrocolorimetric methods, it gives results close to other fluorometric methods such as ORAC [40], and it is widely used on fruit and vegetable matrices. Additionally, in this work, the determination of antioxidant capacity is a complement to the determination of other bioactive compounds by spectroscopy and HPLC. Briefly, the lyophilized sample was mixed with methanol and then centrifuged under the same conditions described for TP determination. Subsequently, 0.1 mL of supernatant was mixed in methanol with 3.9 mL of DPPH (0.030 g/L; Sigma-Aldrich, Steinheim, Germany). Before and after 5 min of storage (until the reaction reached the steady state), the samples’ absorbance was read at 515 nm using the previously described spectrophotometer. All the results were analyzed in triplicate and expressed as milligram Trolox equivalents (TE) per 100 g of dried base (mg TE/100 g db). The percentage of DPPH was calculated following Equation (4):

(4) $$\%\mathrm{DPPH}\;=\frac{\left(A_{control}-A_{sample}\right)}{A_{control}}\times 100$$

where *A_control_* is the absorbance of the control (initial time), and *A_sample_* is the absorbance of the sample after 5 min of storage.

#### 2.4.5. Ascorbic Acid and vitC

The determination of ascorbic acid (AA) and vitC (AA + dehydroascorbic acid (DHA)) was performed using an HPLC-UV detector (Jasco equipment, Cremella, Italy) in duplicate. The determination of AA was based on Igual et al. [41]. Briefly, before injection, the lyophilized sample was extracted with 0.1% oxalic acid (Scharlab, Barcelona, Spain) for 3 min, followed by immediate filtration (0.45 µm). The reduction of DHA to AA using DL-dithiothreitol (Scharlab) as the reductant reagent was used to determine total vitC [42]. Subsequently, 2 mL of dithiothreitol solution (20 g/L) was added to 0.2 g of the sample mixed with 0.3 mL of water. Afterward, the solution was collected to react for 2 h at 25 °C and in darkness, continuing with the same procedure mentioned before for AA determination. An AA standard solution (Sigma-Aldrich, Steinheim, Germany) was used. The instrumentation for the HPLC method was an Ultrabase-C18, 5 µm (4.6 × 250 mm) column (Scharlab), mobile-phase oxalic acid 0.1% (Scharlab), 1 mL/min flow rate, 20 µL injection volume, and 243 nm detection at 25 °C.

#### 2.4.6. In Vitro Digestion

A standardized static IVD method that is suitable for food (COST INFOGEST network) [43,44] was used for sample digestibility evaluation [45]. IVD protocol steps included the following: oral phase (sample and simulated salivary fluid (1:1) with amylase (75 U/mL) at 37 °C, pH 7 for 2 min); gastric phase (oral bolus and simulated gastric fluid (1:1) with pepsin (25,000 U/mL) at 37 °C, pH 3 for 2 h); and intestinal phase (gastric chyme and simulated intestinal fluid (1:1) with pancreatin (800 U/mL) and bile at 37 °C, pH 7 for 2 h); filtration (centrifuging at 4500 rpm at 20 °C for 20 min (Eppendorf Centrifuge 5804 R (Eppendorf); and then filtering through a 1 µm glass microfiber filter [22,45].

The enzyme concentration was estimated according to the activity certified by the manufacturer. The simulated fluids were prepared according to Minekus et al. [43].

All lyophilized samples (TC, PC, SV, and CV) and the blank and soup were subjected to IVD; the samples were collected according to Minekus et al. [43] and Brodkorb et al. [44] and then freeze-dried as explained in Section 2.2. To calculate the in vitro sample digestibility (IVD%), the difference between the initial sample and the undigested one was established; then, it was divided by the initial mass sample and finally multiplied by 100, according to Batista et al. [46], Igual et al. [24], and Igual et al. [25].

All samples (TC, PC, SV, and CV) before and after the IVD were analyzed in triplicate for TotC and L, TP contents, antioxidant activity, and AA and vitC, as explained in Section 2.4.2, Section 2.4.3, Section 2.4.4 and Section 2.4.5, respectively.

Bioaccessibility was determined using Equation (5), proposed by Khouzam et al. [47]:

(5) $$\mathrm{Bioaccessibility}\;=\frac{A}{B}\times 100$$

where *A* is the bioactive compound in the bioaccessible fraction after IVD’s concentration (filtrate after filtration); *B* is the bioactive compound in the sample before digestion’s concentration. A control was also analyzed for correction of the final bioaccessible fraction.

#### 2.4.7. Statistical Analysis

Variability in texture, color, and antioxidant and bioactive content among conditions and undigested and digested samples was analyzed with ANOVA, followed by an LSD post hoc test (Fisher’s least significant difference test) to determine significant differences (*p* < 0.05). The software used was Statgraphics Centurion version 18.1.13 (STSC, Rockville, MD, USA).

A correlation analysis between bioactive compounds and AC was conducted with a 95% significance level, applying the Pearson correlation. The same analysis was also performed on color parameters and TotC content.

## 3. Results and Discussion

### 3.1. Textural Kinetics of Pumpkin and Carrot Pieces to Standardize Cooking

**Figure 1:**
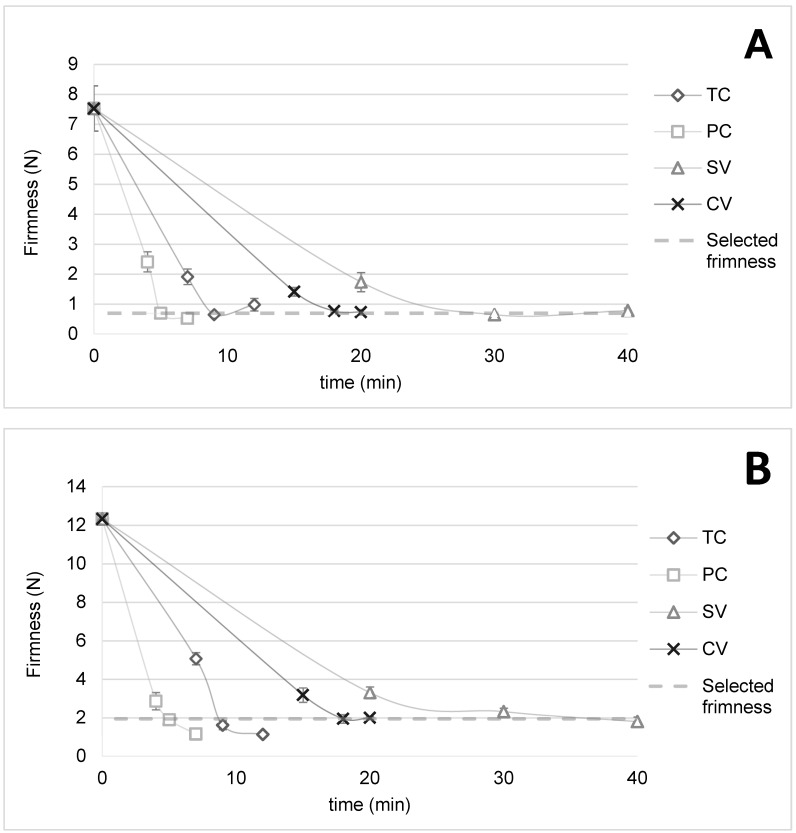
Vegetable firmness at different treatment conditions. Note: Pumpkins (**A**) and carrots (in phloem tissue) (**B**); TC, traditional cooking; PC, pressure cooking; SV, sous-vide; CV, cook-vide.

Carrot and pumpkin firmness were analyzed at varying cooking times in the different cooking treatments at a constant temperature (Figure 1), since the texture was used as a standard to establish comparisons between soup preparation methods to obtain vegetables cooked to a similar degree. As expected, the firmness of both vegetables decreased when the cooking time increased, obtaining significant differences (*p* < 0.05) in the first three times for each treatment.

The tendency observed by Iborra-Bernad et al. [33] and Iborra-Bernad et al. [36] involving the effect of temperature in the softening process being greater than the cooking time was also corroborated. SV and CV samples cooked at 90 °C were firmer than samples cooked with shorter treatments at higher temperatures (TC at 100 °C and PC at 120 °C).

Loss of firmness is associated with substantial dissolution, depolymerization, and, apparently, the destruction of cell wall pectins [48]. The obtained results are in agreement with Iborra-Bernad et al.’s [33] texture of carrots for CV cooked under the same conditions. Furthermore, authors such as Koç et al. [49] observed firmer texture for SV than for CV, although carrots for the SV treatment in those studies were vacuum-sealed without water inside the pouch. That difference should be considered when comparing results, since heat transfer is conducted differently in the presence of water in contact with vegetables, with the heat transfer coefficient of surfaces being higher in boiling water (CV) than in liquid water (SV) [36]. Thus, SV treatment conditions were the mildest.

Iborra-Bernad et al. [33] reported changes in the characteristics of carrot pectic substances under TC (boiling water at 100 °C) by an increase in the β-elimination reaction, causing a slower loss of firmness when increasing cooking time. This effect could also explain the results obtained in the present study.

The selected conditions were established when firmness was 0.690 (0.058) N (standard deviation shown in brackets) in cooked pumpkins and 1.94 (0.29) N in cooked carrots, considering all cooking treatments and the shorter process time. No significant differences (*p* > 0.05) were found in firmness between selected samples cooked with TC, PC, and SV for pumpkins (Table 2). However, only PC and CV presented no significant differences for carrots (*p* > 0.05). This means that although similar final firmness was reached during cooking, it was difficult to obtain a constant level of firmness for both vegetables under the same conditions. The selected conditions were TC for 9 min, PC for 5 min, SV for 30 min, and CV for 18 min.

Instrumental firmness has been reported to correlate with sensory perception [33], providing a quick and cheap tool for establishing optimum cooking time and evaluating the cooking quality of ready-to-use food products [37].

### 3.2. Effect of Cooking Method on the Physical Properties of Soup and Process Yield

#### 3.2.1. Soups’ Physical Properties

The mechanical and optical properties of selected soup vegetables were studied to describe the soup’s physical properties. Texture analysis was conducted on pumpkins and carrots, evaluating their firmness. The results are shown in Table 2.

As explained in Section 3.1, cooking conditions affected vegetables differently, since for carrots, only PC and CV presented no significant differences (*p* > 0.05), whereas for pumpkins, only CV presented a significant difference in their firmness (*p* < 0.05).

Considering the complexity given by the high number and the different nature of this recipe’s ingredients, it was expected that there would be difficulties reaching a fair comparison for the different cooking techniques. However, the selected firmness analysis provided a global vision of the process impact, allowing for a subsequent comparison of the soups’ nutritional and functional properties. Additionally, to decide whether a vegetable is adequately cooked, firmness is one of the main factors considered by consumers [33], so it is a valuable parameter to consider when designing this kind of food.

The cooking method’s impact on soups’ color was studied by comparing raw and cooked samples; pumpkins and carrots were selected for color analysis. Table 3 shows the chromatic coordinates (L*, a*, and b*), total color differences (ΔE), hue (h*), and chroma (c*) obtained for raw pumpkins and carrots before and after cooking under the TC, PC, CV, and SV methods.

The heat treatments caused the colorimetric parameters of raw pumpkins and carrots to be significantly different (*p* < 0.05) from cooked ones (Table 3). As expected, the cooked samples showed lower L* values, and TC and PC presented the significantly highest values (*p* < 0.05), followed by CV and SV for pumpkins and carrots. Although it is reported that the SV method better retains the sample’s color than TC and CV in green bean pods and carrots [33,50], other studies’ results disagree when studying purple-flesh potatoes, pumpkins, and carrots [26,37,51].

After all cooking procedures, redness values (a*) and yellowness values (b*) decreased compared with raw samples. PC showed the highest a* and b* values for pumpkins and carrots and did not show significant differences (*p* > 0.05) with raw carrots. Pressure cooking (PC) has a limited effect on pigments (e.g., chlorophyll, carotenoids, anthocyanins, etc.) responsible for the color of fruits and vegetables [52,53]. The PC treatment can increase the inactivation of enzymes and micro-organisms, which can result in undesired chemical reactions (both enzymatic and non-enzymatic) that affect the color of vegetables [52,53].

Chroma measurements (C*) were highest in raw pumpkins and carrots, which indicates a more vivid color, followed by the PC and TC methods in pumpkins and PC and CV in carrots, with no significant differences (*p* > 0.05) among those cooking conditions (Table 3). It has been reported that vacuum cooking (CV) can preserve the color of raw ingredients better than other cooking procedures [26,37]; this is in agreement with this study’s results only for carrots.

The hue angle (h*) showed no significant differences (*p* > 0.05) between raw, CV, and SV in pumpkins, whereas there were no significant differences (*p* > 0.05) between raw and SV methods in carrots. The same results were reported by Trejo Araya et al. [51] in carrots; the authors also found that cooked samples presented the greatest hue angle, as in this case. Conversely, Gomes da Silva, et al. [54] reported that a hue angle decrease remains for SV in pumpkins.

As a consequence of cooking, lower global color difference values (ΔE* raw) were observed in cooked pumpkins applying TC and PC (*p* < 0.05), whereas lower values were observed in carrots in PC and CV, meaning that the color of products is more similar to the raw samples in those cases. Iborra-Bernad et al. [36] found that the lowest differences in total color difference belong to TC samples compared with CV and SV methods, suggesting that higher temperatures facilitate the destruction of carotenoid–protein complexes, increasing β-carotene extraction by destabilizing cells’ homeostasis.

Comparing the global color difference of cooking techniques to TC (ΔE* TC), significant differences were found (*p* < 0.05). In cooked pumpkins, lower values were found for PC. This would indicate that the color effect generated from cooking was similar to TC, whereas for the rest of the methods, the difference was not significant (*p* > 0.05). For carrots, lower values were also observed in PC and also in CV, being not significantly different (*p* > 0.05).

The difficulty in the comparison between the color parameters obtained and the data reported in the literature should be highlighted [54]. The color of pumpkins and carrots is mainly related to carotenoid pigment content, which is affected by variety, maturity, and growth conditions, causing a high level of variability in the color of these vegetables [54]. In this case, after Pearson’s correlation analysis, it was found that a*, b*, and L* values for carrots were significantly and positively related to the TotC content (*p* < 0.05) of soup samples before IVD, as was expected. However, this correlation was only found for pumpkin in the L* value (*p* < 0.05).

#### 3.2.2. Process Yield

Considering the differences in cooking techniques in terms of temperature, pressure, cooking media state, and material in contact with the sample (Table 1), the process yields were expected to be different. One of the principal characteristics of SV cooking is that there are no mass losses in the sample, since it is vacuum-sealed, meaning that the yield was 100% in this case. During the TC, CV, and PC methods, water evaporation causes the process yield to be lower in all cases. The cooking times differed in each case, as established in the previous section, resulting in yields of 87%, 77%, and 68% for PC, CV, and TC, respectively. The difference in process yield influences the final mass of the product, which must be considered when thinking in production terms. The energy and supplies needed for producing the soup will vary to obtain the same final product’s neat weight, which is a relevant factor to take into account when escalating the process.

As the final water volume contained in the product is not the same for different cooking treatments, the concentration of nutrients will also be different. Moreover, as it has been explained, ingredients’ physical and chemical responses differ when applying the different cooking treatments. To establish the results independent of the final volume of the product, comparisons are made based on dry products.

### 3.3. In Vitro Digestibility

**Figure 2:**
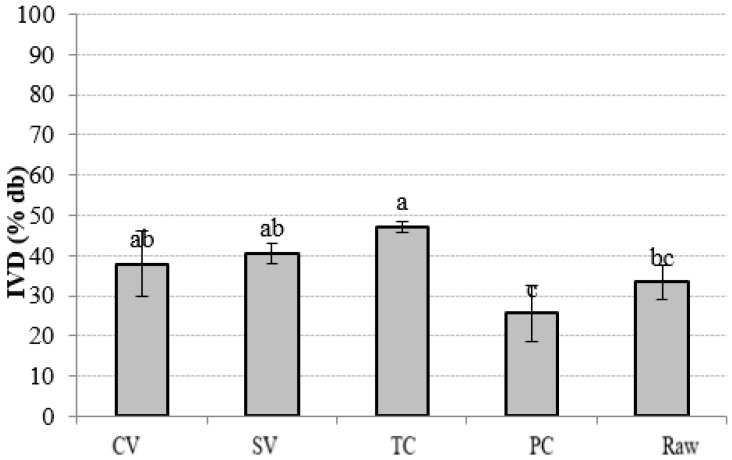
In vitro digestibility of soups cooked by different methods. Note: Mean values and standard deviations (error bars) on a dried base sample. For each cooking method, small letters indicate homogeneous groups established by ANOVA (*p* < 0.05, Fisher’s LSD test). TC, traditional cooking; PC, pressure cooking; SV, sous-vide; CV, cook-vide.

The IVD analysis reproduces the chemical–enzymatic catalysis that occurs in the proximal tract of the monogastric digestive system [55]. Figure 2 shows the IVD% of all soup samples, which was relatively low. The maximum IVD% was reached by TC (47.1% (1.3)) and presented no significant difference (*p* > 0.05) with CV and SV methods. The soups cooked by PC had the lowest IVD% (25.7% (7.0)), presenting significant differences (*p* < 0.05) with raw soup (33.4% (4.1)), which was initially expected to have lower values. Generally, cooking enhances the digestibility of food, so fewer residues are carried to the large intestine [15]. In pulses such as lentils, thermal processing gradually solubilizes pectin at the middle lamella, causing cell separation, and it also increases cell wall permeability. The residual degree of nutrient bioencapsulation is then determined by the overall effect of the cooking process, which affects the accessibility of digestive enzymes and the digestibility patterns of protein and starch [56].

To estimate preabsorptive events, such as the bioaccessibility of nutrients from a food matrix, IVD is also useful [20].

### 3.4. Effect of Cooking Method on the Bioactive Compounds of Soup and Their Bioaccessibility

Soups’ principal ingredients, in addition to water, are pumpkins (9%), carrots (8%), and lentils (8%). The quantity and nature of bioactive compounds provided by the soup will primarily be a consequence of the release of these pulses and vegetables.

To follow the differences between cooking techniques and characterize the soups’ bioactive potential, TotC, TPs, and vitC were measured as indicators. These compounds have been selected when studying vegetables, pulses, and soups for being chemically hydrophobic and hydrophilic, respectively, and sensitive to both temperature and oxygen [5,11,14,36,54]. Lycopene and ascorbic acid were also measured. In the diet, the sum of the recommended daily value of vitamins C, E, and β-carotene is 72 mg/day [57]. However, there is no specific recommendation (RDI) for total carotenoids and phenols.

In the current study, the term bioaccessibility refers to the fraction of bioactive compounds previously mentioned that were released from the soup during IVD and therefore accessible for absorption in the organism [18,22]. Bioaccessibility studies provide relevant information regarding interactions between food components and their nutrients, as well as the effect of pH and enzymes and food preparation and processing practices on potential nutrient absorption [58]. Food processing is one of the main determining factors for estimating bioaccessibility, because it can either increase or decrease the bioaccessibility of nutrients and bioactive compounds [20]. Functional and structural properties of vitamins, phenolic compounds, and carotenoids may be altered by changes in the food environment, such as heat and pH. Consequently, the bioaccessibility of these compounds could also be influenced by these factors [59]. The bioaccessibility results are presented in Table 4 and discussed in further detail in each corresponding section. In general, phenolic compounds showed higher bioaccessibility values, followed by L and finally TotC; this tendency was observed for all cooking techniques and raw samples.

Soups’ number of ingredients, proportion, and different nature make it difficult to directly compare the obtained results with a bibliography. To our knowledge, the phytonutrient bioaccessibility of vegetables and legumes cooked under SV and CV cooking has not been evaluated. Moreover, not many studies have reported vegetable and legume accessibility under the same conditions as those presented in our study. Additionally, it should be considered that degradation of the cell wall and membranes caused by cooking has been reported to favor the release of carotenoids and phenolic compounds during digestion or, on the contrary, cause other macromolecules to entrap them [60]; consequently, the results could vary between matrixes and conditions.

Evaluating ingredients separately, some studies have reported 3–6% of TotC bioavailability in raw carrots [20,60]; in particular, β-carotene bioavailability of 2.5–29% was raised when cooking techniques such as boiling and PC were applied (4–30%) [20,61]. For raw pumpkins, 10–15% bioavailability values were reported, barely rising when boiled (11–19%) [20,61]. TP compound bioaccessibility in raw carrots has been reported to reach 13% [60]; our results in soup were higher.

When comparing phytonutrient bioaccessibility in other food matrixes, fresh and pasteurized orange juice present similar values for TP (10–20%) [20,62]. In the case of L, in raw tomatoes, it reaches 5% and increases to 10% after blanching [20]; similar results were obtained for soups in our study. TotC in fresh legume mixtures such as green gram and amaranth leaves and chickpea and amaranthus mixtures had a bioaccessibility of 3%, rising to 5% when dry heat is applied [20].

VitC and AA were not detected in digested samples, so their bioaccessibility was not presented in Table 4. VitC (in L-AA and L-DHA forms) is a water-soluble vitamin sensitive to thermal heating. As a consequence, cooking losses were expected, but it has also been reported to have significant decreases in bioaccessibility after gastrointestinal digestion [63], which corresponds with our results.

#### 3.4.1. TotC and L

The vegetables’ tissues will be affected differently depending on the cooking method used. A significant increase (*p* < 0.05) in energy is necessary for the rupture of the dense cell walls and the dissolution of pectin. Cooking at a higher temperature for a more extended period should cause more tissue damage, leading to better carotenoid extraction [64], since it could lead to the most effective release of TotC compared with the other processes [37]. Another study has shown that different-sized pieces of vegetables affect carotenoids’ release from the tissue, influencing their extraction [65].

**Figure 3:**
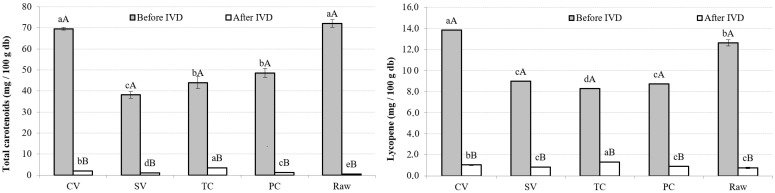
Total carotenoids and lycopene in different treatment conditions, before and after IVD. Note: Mean values and standard deviations (error bars) of total carotenoids and lycopene content in different cooking technique samples. For each cooking method, capital letters indicate homogeneous groups established by ANOVA (*p* < 0.05, Fisher’s LSD test) by comparing it before and after in vitro digestion (IVD). Small letters indicate homogeneous groups established by ANOVA (*p* < 0.05) by comparing samples before and after IVD between cooking methods. db, dry weight basis.

In the present study, before digestion, higher total carotenoid values were obtained in the CV method and raw samples, with no significant differences (*p* > 0.05) between them, followed by PC and TC, and finally SV. L content was higher in CV than in raw samples (Figure 3).

It is reported that the analysis of carotenoids is affected by the variation in their quantity among vegetables, the existence of many carotenoids, and the nonuniform distribution of carotenoids between samples [66]. Considering the mixture of ingredients in the soups, it is difficult to compare the TotC and L determinations with the reported contents of isolated vegetables.

Rinaldi et al. [37] concluded that in pumpkins, cooking procedures significantly increase (*p* < 0.05) almost all the carotenoid contents compared with the raw vegetable, especially vacuum cooking. However, it must be considered that in the present study, vegetables were in contact with water, so the effect of vacuum on the tissues will differ. Torres de Castro et al. [64] did not find differences in carotenoid content after boiling, steaming, and steam microwaving in carrots compared with raw samples.

Significantly different results (*p* < 0.05) were obtained when comparing the total carotenoid values and L values before and after digestion. IVD caused a significant decrease (*p* < 0.05) in TotC and L composition, including the raw sample. Carotenoid digestibility is highly dependent on food matrix characteristics such as the presence of other carotenoids, dietary fiber and fat, and food processing [67]. As explained, thermal treatment during cooking softens the cell structure, so the digestive enzymes can work more efficiently; this may cause the release of carotenoids from the food matrix [68]. It also inactivates oxidative enzymes and denatures the carotenoid–protein complexes that exist in vegetable cells [69]. Additionally, it was reported that heating has a positive effect on the micellization of carotenes in carrots [70]. However, during gastric digestion, the presence of dietary fiber reduces carotenoid micellization, necessary for its further absorption, as fluids become soluble in the gel [20]. This may explain the relatively low TotC and L values obtained after IVD.

When comparing cooking techniques, significant differences were found (*p* < 0.05); TC reached the maximum TotC and L content after IVD, although before the digestion process, it did not show the highest value. Additionally, the raw sample presented lower TotC and L content after IVD, although it had the highest values before digestion. These results indicate the importance of digestion process studies to better compare cooking techniques.

In agreement, TotC and L’s highest bioaccessibility was found in TC (Table 4), as for all studied bioactive compounds. That method was followed by SV and PC, with no significant differences (*p* > 0.05), and then CV. The lowest bioaccessibility was evident in the raw sample. The bioaccessibility of carotenoids depends on the formation of micelles, as they are responsible for transporting liposoluble compounds to the intestine [20]. Rodríguez-Roque et al. [59] reported that the fiber presence negatively affects the bioaccessibility and absorption rate of carotenoids. Food processing methods soften cell walls and disrupt protein–carotenoid complexes, increasing carotenoid release and its bioaccessibility [71].

Considering that soup consists of a hydrophilic matrix, the extraction of the nonpolar compounds would be limited. A proven strategy to effectively improve the bioaccessibility of TotC and L could be adding lipids to the system so they may function as nonpolar solvents, increasing liberation [70,71,72,73].

#### 3.4.2. TP

**Figure 4:**
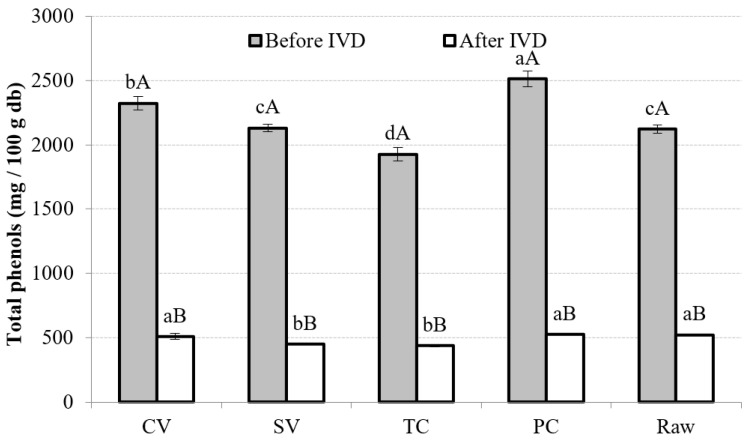
Total phenols in different treatment conditions, before and after IVD. Note: Mean values and standard deviations (error bars) of total phenolic content in different cooking technique samples. For each cooking method, capital letters indicate homogeneous groups established by the ANOVA (*p* < 0.05, Fisher’s LSD test) by comparing it before and after in vitro digestion (IVD). Small letters indicate homogeneous groups established by ANOVA (*p* < 0.05) by comparing samples before and after IVD between cooking methods. db, dry weight basis.

Significantly lower values (*p* < 0.05) were found in TP content after IVD for all treatments. Significantly higher TP values were obtained by PC before IVD, followed by CV, SV, raw, and finally TC. After IVD, PC, CV, and raw samples presented no significant differences (*p* > 0.05) (Figure 4).

The lower values obtained after IVD could be explained by the pH changes in the digestive process causing a decrease in the amount of TPs, since these compounds undergo several chemical reactions, mainly oxidation and polymerization, forming other phenolic derivatives, such as chalcones, which are less soluble, affecting their determination [59,62]. Moreover, the interaction with enzymes and minerals, dietary fiber, or proteins released during digestion might affect polyphenols’ solubility and bioaccessibility [59,74].

However, during gastrointestinal digestion, polyphenols have detached from their carbohydrate moiety, which could result in higher determinations of polyphenol content [20]. In addition, the existing information is frequently contradictory, since under the same cooking conditions, some vegetables experience a decrease in the TP content, whereas others experience an increase [75]. Differences between results could be explained by the fact that depending on the food matrix, phenolic constituents may display antagonistic or synergistic interactions among themselves or with other substances [37].

In the current study, TP bioaccessibility was higher for the raw sample (Table 4), followed by TC, then SV, CV, and PC; all treatments presented significant differences (*p* < 0.05). None of the cooking techniques were able to release phenols from the matrix. Other studies have reported that the interaction between phenols, macronutrients, and dietary fibers, involving covalent bonds and noncovalent hydrophobic interactions, might reduce TP’s solubility and availability, influencing its bioaccessibility during digestion [59,62,76].

#### 3.4.3. AA and vitC

**Figure 5:**
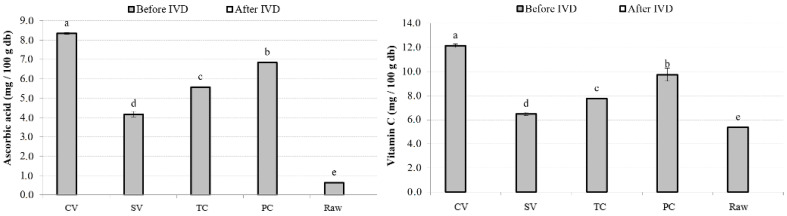
Ascorbic acid and vitamin C in different treatment conditions. Note: Mean values and standard deviations (error bars) of ascorbic acid and vitamin C in different cooking technique samples expressed as mg of β-carotene per 100 g of dried base sample. Small letters indicate homogeneous groups established by ANOVA (*p* < 0.05, Fisher’s LSD test). db, dry weight basis.

VitC is ingested in both reduced (AA) and oxidized (DHA) forms. In the present study, AA and vitC were measured in samples cooked under the different treatments before and after IVD. Results were significantly different before IVD; the following tendency was the same for vitC and AA: the higher values were obtained when cooking under the CV method, then PC, TC, SV, and finally the raw sample (Figure 5). The presence of DHA explains the difference between AA and its values. As was expected, after Pearson’s correlation analysis, AA was significantly and positively related to the vitC content (0.9288, *p* < 0.05) of samples.

Raw samples were expected to have higher values than treated samples, since AA and DHA are sensitive to temperature, oxygen exposure, and light, which makes their retention difficult during cooking [5]. Consequently, all treatments should have caused a decrease in these compounds. It has been reported that different cooking methods caused losses of approximately 50% for AA when compared with uncooked pumpkin samples [54]. In the current study, however, the results in the raw samples were lower, which could be attributed to the early oxidation of the samples.

After IVD, no values were detected for either sample, meaning that vitC was unstable after digestive conditions. The same results were reported by Rodriguez Roque et al. [59], who suggested that alkaline pH, temperature, oxygen, light, and the enzyme activity inherent to in vitro gastrointestinal digestion could enhance vitC oxidation or complex formation with other constituents. AA and vitC bioaccessibility were not calculated, since they were not detected in the digested samples.

#### 3.4.4. AC

Generally, the presence of diverse chemical compounds could influence the antioxidant activity; the synergistic or antagonistic effects of these compounds play a crucial role in the resulting measurements [77]. Samples’ antioxidant activity depends on the composition and concentration of antioxidants, such as vitamins, phenols, and carotenoids, among many other factors [78]. The antioxidant activities of cooked samples in this study were significantly and positively related to vitC (0.7715, *p* < 0.05) and AA (0.8609, *p* < 0.05). Difficulties are posed in comparing these results with other reports because of the complexity of the matrix. Total antioxidant activity cannot be explained simply by the sum of its components; therefore, other possible interactions between them must be contemplated [79]. When comparing pumpkins with other vegetables, Hagos et al. [80] also found that the concentrations of AA in pumpkins, TPs, and total flavonoids correlate significantly with antioxidant activity. In broccoli samples, positive correlations were found between total AA content and antioxidant activity [79]. In the case of berries, vitC has been reported to strongly affect antioxidant activity [77].

**Figure 6:**
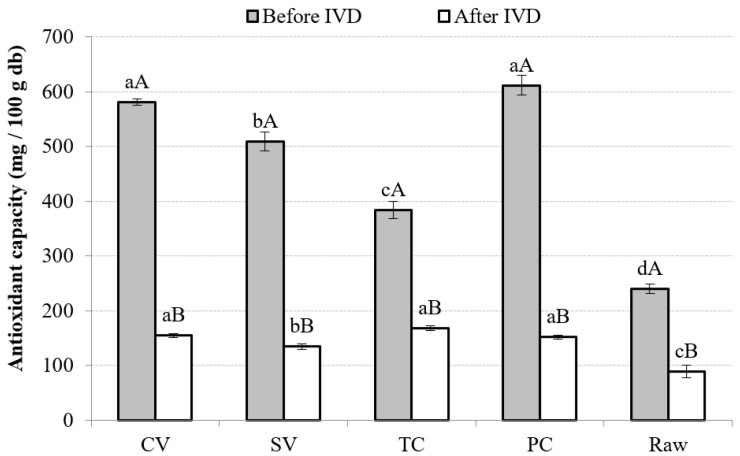
Antioxidant capacity in different treatment conditions, before and after IVD. Note: Mean values and standard deviations (error bars) of antioxidant capacity in different cooking technique samples. Results are expressed as milligram Trolox equivalents per 100 g of dried base (mg TE/100 g db). For each cooking method, capital letters indicate homogeneous groups established by ANOVA (*p* < 0.05, Fisher’s LSD test) by comparing it before and after in vitro digestion (IVD). Small letters indicate homogeneous groups established by ANOVA (*p* < 0.05, Fisher’s LSD test). db, dry weight basis.

The DPPH method was used to assess the AC of the samples. Figure 6 shows that the PC and CV methods had higher antioxidant activity before IVD, presenting significant differences (*p* < 0.05) with the rest of the cooking techniques. However, after IVD, PC, CV, and TC showed higher values. Raw samples had the lowest values in both stages, showing that thermal treatment improves the antioxidant activity of the soups measured by this method.

## 4. Conclusions

It is difficult to reach a unique conclusion about the preferred soup-cooking method, since they all have advantages and disadvantages regarding process yield, the sample’s physical properties, and bioactive compounds’ bioaccessibility.

In the current study, the treatment conditions were set based on the bibliography for reaching the same final vegetable texture. However, to preserve nutritional and bioactive compounds, it is necessary to determine the optimal cooking conditions for each method, considering its bioaccessibility.

The heat treatments induced colorimetric alterations in pumpkins and carrots for all treatments. Reductions in redness values (a*) and yellowness values (b*) were observed in comparison to raw samples. In particular, PC exhibited the highest a* and b* values for both pumpkins and carrots. Prior to digestion, the highest total carotenoid values were observed in the CV method and raw samples, with no statistically significant differences between them, followed by PC and TC, and lastly SV. Lycopene content was notably higher in CV than in raw samples. PC yielded higher TP values, followed by CV, SV, raw, and TC, respectively. After in vitro digestion (IVD), significantly diminished values were observed in total carotenoids, lycopene, and total phenol content for all treatments.

This study’s results demonstrate the importance of considering the digestion process when comparing cooking techniques. In the future, further research should be conducted on structural changes during cooking and their effect on bioactive compounds’ bioaccessibility. Additionally, it would be necessary to continue studies exploring both the sensory profile of the product and consumer perception.

## Figures and Tables

**Table 1:** Cooking technique comparison: boiling (traditional cooking), pressure cooking, sous-vide, and cook-vide.

	TC	PC	SV	CV
Cooking temperature	100 °C	120 °C	90 °C	90 °C
Cooking time	9 min	5 min	30 min	18 min
Material in contact with the sample and cooking media state	Sample in contact with boiling water	Sample in contact with heating boiling water	Sample inside of a vacuum-sealed pouch surrounded by liquid hot water	Sample in contact with boiling water

Note: Traditional cooking (TC), pressure cooking (PC), and two vacuum-cooking treatments: sous-vide (SV) and cook-vide (CV).

**Table 2:** Vegetable firmness after applying different cooking techniques.

**Vegetable**	**Cooking Method**	**Firmness (N)**
Pumpkins	TC (9 min)	0.65 (0.07) ^b^
	PC (5 min)	0.70 (0.08) ^b^
	SV (30 min)	0.64 (0.03) ^b^
	CV (18 min)	0.77 (0.03) ^a^
Carrots	TC (9 min)	1.6 (0.2) ^c^
	PC (5 min)	1.90 (0.18) ^b^
	SV (30 min)	2.3 (0.2) ^a^
	CV (18 min)	1.94 (0.18) ^b^

Note: Mean values with standard deviations are shown in brackets. For each vegetable, significant differences between cooking techniques (*p* < 0.05, Fisher’s LSD test) are presented with different letters. TC, traditional cooking; PC, pressure cooking; SV, sous-vide; CV, cook-vide.

**Table 3:** Vegetable color of raw and cooked samples.

Vegetable	Cooking Method	a*	b*	L*	C*	h*	ΔE* Raw	ΔE* TC
Pumpkins	Raw	19 (1.9) ^a^	45 (3.7) ^a^	51.5 (1.67) ^a^	49 (4.1) ^a^	1.18 (0.020) ^b^		18.2 (3.22) ^a^
	TC	10.9 (0.47) ^c^	35.4 (0.90) ^b^	38.3 (1.43) ^b^	37.1 (0.94) ^b^	1.271 (0.0103) ^a^	18.04 (1.023) ^b^	
	PC	12.6 (0.45) ^b^	37 (3.0) ^b^	39.3 (1.08) ^b^	39 (2.9) ^b^	1.24 (0.027) ^a^	16.0 (1.48) ^b^	3.7 (1.26) ^c^
	SV	11 (1.8) ^c^	26 (4.8) ^c^	34 (3.1) ^d^	28 (4.9) ^c^	1.16 (0.050) ^b^	27 (3.7) ^a^	11 (3.2) ^b^
	CV	9.27 (1.68) ^d^	27 (3.6) ^c^	36.0 (1.70) ^c^	29 (3.0) ^c^	1.24 (0.064) ^b^	25 (2.9) ^a^	9 (3.3) ^b^
Carrots	Raw	28 (3.0) ^a^	36 (3.1) ^a^	48 (2.0) ^a^	45 (3.7) ^a^	0.91 (0.042) ^b^		14.7 (1.94) ^b^
	TC	20 (1.9) ^b^	30 (4.2) ^b^	38 (2.5) ^c^	36 (3.7) ^c^	0.99 (0.077) ^a^	15 (2.9) ^b^	
	PC	20.7 (1.53) ^b^	34 (4.6) ^a^	40.9 (0.60) ^b^	40 (4.4) ^b^	1.02 (0.052) ^a^	11 (2.4) ^c^	6 (2.2) ^c^
	SV	14.9 (1.40) ^c^	18 (2.6) ^c^	26.8 (1.71) ^d^	23 (2.4) ^d^	0.88 (0.075) ^b^	31 (1.7) ^a^	18 (1.9) ^a^
	CV	21.4 (0.84) ^b^	35.9 (1.84) ^a^	39.1 (1.14) ^c^	41.8 (1.67) ^b^	1.03 (0.028) ^a^	11.60 (1.139) ^c^	6.2 (1.64) ^c^

Note: Mean values with standard deviations are shown in brackets. For each vegetable, significant differences between cooking techniques (*p* < 0.05, Fisher’s LSD test) are presented with different letters. The measurement was obtained eight times. ΔE* raw: total color differences between raw soup and after applying different cooking techniques; ΔE* TC: total color differences between TC and different cooking techniques. TC, traditional cooking; PC, pressure cooking; SV, sous-vide; CV, cook-vide.

**Table 4:** Bioactive compounds’ bioaccessibility in all samples.

	TotC Bioaccessibility (%)	Lycopene Bioaccessibility (%)	TP Bioaccessibility (%)
CV	2.84 (0.14) ^b^	7.5 (0.5) ^c^	21.86 (1.02) ^c^
SV	2.83 (0.05) ^b^	9.28 (0.15) ^b^	21.01 (0.13) ^d^
TC	8.00 (0.14) ^a^	15.8 (0.4) ^a^	22.7 (0.4) ^b^
PC	2.63 (0.07) ^b^	10.2 (0.3) ^b^	20.89 (0.15) ^d^
Raw	0.78 (0.16) ^c^	6.1 (0.6) ^d^	24.52 (0.17) ^a^

Note: Mean values with standard deviations are shown in brackets. For each parameter, significant differences between cooking techniques (*p* < 0.05, Fisher’s LSD test) are presented with different letters. TotC, total carotenoids; TP, total phenols.

## Data Availability

The original contributions presented in the study are included in the article, further inquiries can be directed to the corresponding authors.
